# Hypertriglyceridemia‐modulated gut microbiota promotes lysophosphatidylcholine generation to aggravate acute pancreatitis in a TLR4‐dependent manner

**DOI:** 10.1002/imt2.70003

**Published:** 2025-02-11

**Authors:** Xiaofan Song, Lei Qiao, Xina Dou, Jiajing Chang, Xiaonan Zeng, Tianjing Deng, Ge Yang, Peiyun Liu, Cheng Wang, Qinhong Xu, Chunlan Xu

**Affiliations:** ^1^ School of Life Sciences Northwestern Polytechnical University Xi'an China; ^2^ Xianghu Laboratory Hangzhou China; ^3^ Department of Geriatric Surgery the First Affiliated Hospital of Xi'an Jiaotong University Xi'an China

## Abstract

Hypertriglyceridemia (HTG) can lead to the disorder of gut microbiota in mice, resulting in the increase of endotoxin content. HTG can also aggravate the damage of intestinal barrier function and intestinal bacterial translocation in acute pancreatitis (AP) mice. Toll‐like receptor 4 gene *(Tlr4)* knockout can significantly reduce gut permeability and endotoxin invasion in AP mice. In addition, HTG‐modulated gut microbiota could up‐regulate glycerophospholipid metabolism and increase lysophosphatidylcholine (LysoPC) content in a TLR4‐dependent manner, thereby aggravating pancreatic injury in AP.

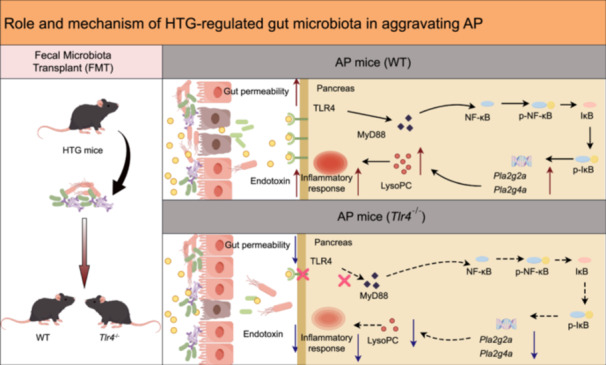

## ETHICS STATEMENT

The ethics application (No. 20220721) was approved by the Ethics Committee of Northwestern Polytechnical University, China.

To the Editor,

Acute pancreatitis (AP) is a common critical emergency of the digestive system, which is an acute inflammatory injury of the exocrine system of the pancreas caused by gallstones, alcohol and high triglycerides [1]. AP cannot only lead to pancreatic exocrine insufficiency but also increase the risk of chronic pancreatitis and seriously affect the quality of life of patients [2].

Hypertriglyceridemia (HTG), as a major public health problem, has a prevalence of 25%–50% worldwide [3]. Currently, HTG‐induced AP is rising in incidence, now ranking as the third most common cause of the disease. [4]. The patients with HTG‐induced pancreatitis have a higher risk of multiple organ failure and pancreatic necrosis [5]. Animal studies suggest that the accumulation of free fatty acids (FFAs) produced by triglyceride hydrolysis and excessive activation of inflammatory responses may contribute to the pathogenesis of AP [6].

Recently, the role of gut microbiota as a crucial mediator in the development of AP has gained increasing attention, particularly how its dysregulation is closely linked to pancreatic injury. [7]. Disruption of the gut epithelial barrier and increased gut permeability, including alterations in tight junction proteins, are frequently observed in AP pathology [8]. Increased gut permeability leads to the translocation of some gut bacteria including *Staphylococcus*, *Enterococcus*, *Escherichia coli*, and *Klebsiella* into the pancreas, which aggravates the local inflammatory state and mucosal immune dysfunction [9]. The composition of the gut microbiota in the hypertriglyceridemic acute pancreatitis (HTG + AP) patients was significantly correlated with prognostic markers, including disease severity, local and systemic complications, intensive care unit admission, and mortality [10]. Furthermore, a study of 1141 subjects categorized into control and abnormal groups based on blood lipid tests found significantly lower levels of *Oscillospira* and *Anaerostipes* in HTG patients, with a strong negative correlation to triglyceride (TG) levels. *Prevotella*, *Fusobacterium*, *Megamonas*, *Megasphaera* and *Acidaminococcus* were significantly increased in HTG patients [11]. However, the role and exact mechanisms of the gut microbiota in the exacerbation of AP exacerbated by HTG remain unclear. This study starts with the gut microbiota to explore the mechanisms by which HTG exacerbates AP and its potential targets, which will provide a basis for the clinical diagnosis and treatment of HTG + AP.

## RESULTS AND DISCUSSION

### HTG can aggravate the pancreas injury and gut barrier dysfunction of AP in a gut microbiota‐dependent manner

**Figure 1:**
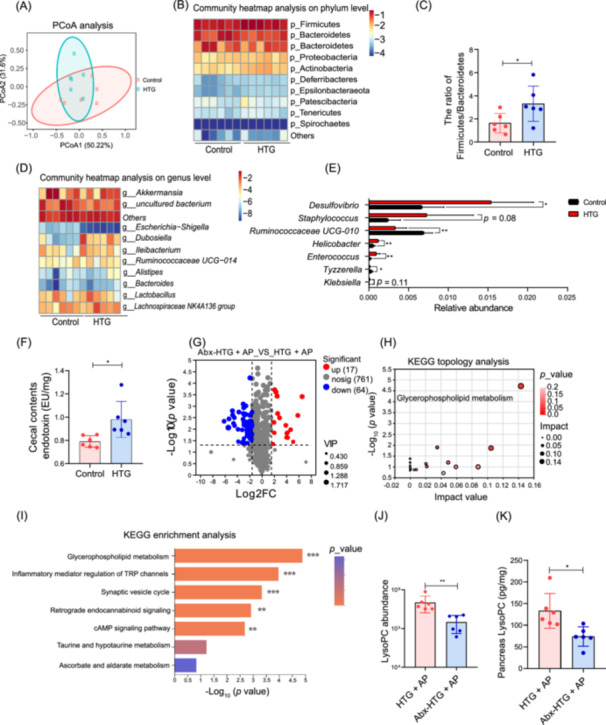
HTG‐modulated gut microbiota influences glycerophospholipid metabolism in HTG + AP mice. (A) Principal component ordination analysis (PCoA) of gut microbiota composition (*n* = 6). (B) Heatmap of gut microbial community at the phylum level (*n* = 6). (C) Ratio of Firmicutes to Bacteroidetes in gut microbiota (*n* = 6). (D) Heatmap of gut microbial community at the genus level (*n* = 6). (E) Differential genus between the HTG and Control groups (*n* = 6). (F) Endotoxin contents in cecal of mice. (G) Volcano plot showing differentially abundant serum metabolites (*n* = 6). (H) KEGG topology analysis of differentially enriched metabolites in mice (*n* = 6). (I) KEGG enrichment analysis of differential metabolites in mice (*n* = 6). Relative abundance of LysoPC in serum (*n* = 6) (J) and pancreas (K). *p < 0.05, **p < 0.01. Abx, antibiotic treatment; HTG + AP, hypertriglyceridemic acute pancreatitis; KEGG, Kyoto Encyclopedia of Genes and Genomes; LysoPC, lysophosphatidylcholine.

HTG + AP mice model was induced by the combination of caerulein and Poloxamer‐407 (500 mg/kg body weight) (Figure S1A,B). The levels of serum TG, total cholesterol (TC) and FFAs were significantly increased in the HTG and HTG + AP mice compared with the Control group (Figure S1C–F). HTG aggravated the pancreatic injury of AP (Figure S1G–J), which is correlated with increased systemic inflammation in the HTG + AP group compared with the AP group (Figure S1K,L). We also observed that HTG aggravated AP‐induced gut barrier dysfunction (Figure S2A–D). HTG + AP mice exhibited elevated rates of bacterial culture and bacterial abundance in both blood and pancreas compared with AP mice. However, this phenomenon was not observed in the blood and pancreas of Control and HTG mice (Figure S2E–G). In addition, α‐diversity analysis showed that abundance‐based coverage estimator index and observed_species were significantly increased in HTG mice compared with Control mice (Figure S3A–D). β‐diversity analysis revealed significant differences in gut microbiota composition between different groups (*p* = 0.027) (Figure 1A). Relative abundance analysis at the phylum level revealed that the HTG mice displayed a higher ratio of Firmicutes/Bacteroidetes compared to the Control group (Figure 1B,C). At the genus level, a profound bloom of pathogenic bacterium including *Desulfovibrio*, *Staphylococcus*, *Helicobacter*, *Enterococcus* and *Klebsiella* was observed in HTG mice. At the same time, the abundance of potentially beneficial bacteria including *Ruminococcaceae UCG‐010* and *Tyzzerella* was reduced in HTG mice (Figure 1D,E). Increased endotoxin content was observed in the cecum of HTG mice (Figure 1F), which may be the consequence of gut microbiota disturbance caused by HTG. To prove that HTG‐modulated gut microbiota is an important factor of aggravating gut barrier damage in HTG + AP, mice were treated with a cocktail of antibiotics to deplete gut microbiota (Figure S4A). The significant reduction in α diversity of the gut microbiota was observed in antibiotic treatment (Abx)‐HTG + AP group (Figure S4B–D). The depletion of gut microbiota effectively alleviated the histological damage, inflammatory response and lipid levels in the pancreas of HTG + AP mice (Figure S4E–M). Gut permeability was attenuated with gut microbiota depletion (Figure S5A–G). A fecal microbiota transplantation (FMT) experiment was performed to analyze the effect of HTG‐modulated gut microbiota on pancreatic injury of AP (Figure S6A). HTG‐modulated gut microbiota caused more severe pancreatic injury and inflammatory response in mice (Figure S6B–J). In addition, HTG‐modulated gut microbiota caused more severe gut barrier impairment and bacteria translocation (Figure S7). Bacteria translocation has been reported in both animal and clinical study, which is often accompanying AP and is believed to be linked to patient outcome [12]. A prior study demonstrated that the western diet aggravated experimental AP by increasing bacterial dissemination [13]. In addition, it has been shown that a high‐fat diet can cause mitochondrial dysfunction, leading to increased oxygen concentration in the gut, which promotes the proliferation of aerobic bacteria such as *Escherichia coli* [14]. These results suggest that HTG aggravates the damage of gut barrier function and bacteria translocation induced by AP by causing gut microbiota disorder, thereby aggravating pancreatic injury in AP mice.

### HTG‐modulated gut microbiota regulates glycerophospholipid metabolism and lysophosphatidylcholine content

Gut microbiota depletion in HTG + AP mice mainly led to the upregulation of 17 metabolites and the downregulation of 64 metabolites (*p* < 0.05 and FC ≥ 3) (Figure 1G). The differential metabolites caused by gut microbiota depletion were enriched in different pathways. Among these pathways, glycerophospholipid metabolism is a top pathway altered in Abx‐HTG + AP mice (Figure 1H,I). We further analyzed the metabolites involved in glycerophospholipid metabolism and found that depletion of HTG‐modulated gut microbiota significantly reduced the level of lysophosphatidylcholine (LysoPC) in serum and pancreas (Figure 1J,K). Phospholipase A2 (PLA2) is the rate‐limiting enzyme in the formation of inflammatory mediators such as LysoPC and is widely involved in inflammatory responses [15]. In this study, we found that depletion of HTG‐modulated gut microbiota resulted in a decrease in serum PLA2 content. The mRNA levels of phospholipase A2 group IIA (*Pla2g2a*) and phospholipase A2 group IVA (*Pla2g4a*) in the pancreas were also downregulated (Figure S8A,B). In addition, FMT showed that mice receiving HTG‐modulated gut microbiota had significantly increased LysoPC content in serum and pancreas, as well as significantly increased serum PLA2 content and *Pla2g2a* and *Pla2g4a* mRNA levels in pancreas (Figure S8C–F). A previous study showed that activation of toll‐like receptor 4 (TLR4) can enhance the content of PLA2 [16], while endotoxin, as a ligand for TLR4, can lead to activation of TLR4. The current results showed that depletion of HTG‐regulated gut microbiota significantly reduced endotoxin content in cecal contents and pancreas, and suppressed TLR4 signaling (Figure S8G–I). In addition, HTG‐modulated gut microbiota resulted in elevated endotoxin content in cecal contents and pancreas and activation of TLR4 signaling in pancreas (Figure S8J–L). A recent study showed that high‐fat diet caused a significant upregulation of glycerophospholipid metabolism and increased serum LysoPC levels in mice. Gut microbiota depletion could restore serum LysoPC levels and decrease glycerophospholipid metabolism in mice fed high‐fat diet [17]. These results indicated that there was a potential interaction between HTG‐modulated gut microbiota and LysoPC formation.

### The regulatory effects of HTG‐modulated gut microbiota on LysoPC content via TLR4 signaling pathway

**Figure 2:**
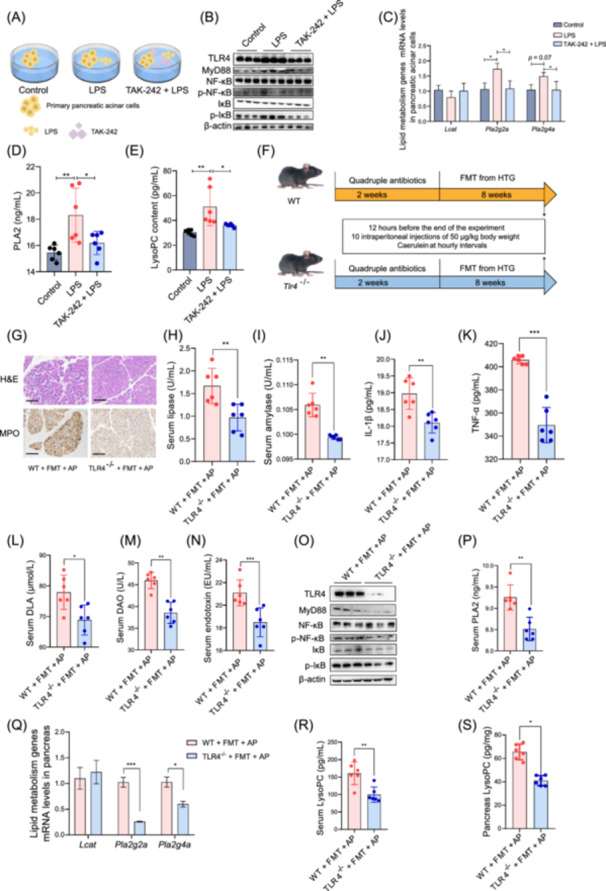
TLR4‐dependent regulation of LysoPC content by HTG‐modulated gut microbiota. (A) Experimental scheme for cell studies. Primary pancreatic acinar cells were treated with LPS or LPS plus TAK242. (B) Effect of LPS and TAK‐242 on the TLR4 signaling pathway in primary pancreatic acinar cells determined by western blot analysis (*n* = 3). (C) Expression of *Lcat*, *Pla2g2a* and *Pla2g4a* genes determined by qRT‐PCR (*n* = 6). (D) Intracellular PLA2 content (*n* = 6). (E) Intracellular LysoPC content (*n* = 6). (F) Schematic illustration of the treatment of C57BL/6 mice (*n* = 10). (G) Representative microscopic features and immunohistochemical staining of MPO in the pancreas of mice (*n* = 3, Scale bars: 100 µm). (H) Serum lipase activity (*n* = 6). (I) Serum amylase activity (*n* = 6). Serum IL‐1β (J) and TNF‐α (K) levels (*n* = 6). Serum d‐LA (L), DAO (M) and endotoxin (N) contents in mice (*n* = 6). (O) The effect of HTG‐modulated gut microbiota on TLR4 signaling pathway between WT and *Tlr4* ^−/−^ mice. (P) The content of PLA2 in serum (*n* = 6). (Q) Lipid metabolism‐related genes mRNA levels in pancreas (*n* = 6). The LysoPC content in serum (R) and pancreas (S) (*n* = 6). *p < 0.05, **p < 0.01. LysoPC, lysophosphatidylcholine; PLA2, phospholipase A2; *Pla2g2a*, phospholipase A2 group IIA; *Pla2g4a*, phospholipase A2 group IVA; *Lcat*, lecithin cholesterol acyltransferase; TLR4, toll‐like receptor 4; MyD88, myeloid differentiation primary response protein 88; NF‐κB, nuclear factor kappa‐B; p‐NF‐κB, phospho‐nuclear factor kappa‐B; IκB, inhibitor of nuclear factor kappa‐B; p‐IκB, phospho‐inhibitor of nuclear factor kappa‐B.

Although many studies have proved the regulatory effects of gut microbiota on glycerophospholipid metabolism and LysoPC, the underlying mechanisms remain unclear [18]. To explore the mechanism between HTG‐modulated gut microbiota and LysoPC formation, the primary pancreatic acinar cells of mice were isolated and pretreated with TAK‐242 (a TLR4 antagonist) followed by lipopolysaccharides (LPS) treatment (Figure 2A). We found that TLR4 was activated, and *Pla2g2a* and *Pla2g4a* mRNA levels, PLA2 content and LysoPC content were significantly increased in primary pancreatic acinar cells exposed to LPS, which could be alleviated by TAK‐242 pretreatment (Figure 2B–E). In addition, we further explored the effects of HTG‐modulated gut microbiota on *Tlr4* ^−/−^ mice (Figure 2F). As expected, *Tlr4* ^*−/−*^ mice showed significantly reduced pancreatic injury and myeloperoxidase (MPO) expression level, reduced inflammatory response and enhanced gut barrier function compared with WT mice (Figure 2G–N). In addition, we observed the inhibition of TLR4 in *Tlr4* ^*−/−*^ mice, as well as a decrease in serum PLA2 content and *Pla2g2a* and *Pla2g4a* mRNA levels and a decrease in LysoPC content in serum and pancreas (Figure 2O–S). A previous study found that lipid release from PLA2 hydrolysis was significantly impaired after TLR4 knockdown in LPS‐stimulated macrophages, inhibiting the production of pro‐inflammatory lipid mediators [16]. As observed in the study, TLR4 knockdown can significantly reduce the content of PLA2 and decrease the synthesis of LysoPC. These results suggest that the HTG‐modulated gut microbiota regulates LysoPC synthesis in AP mice in a TLR4‐dependent manner.

### LysoPC aggravates pancreatic injury and inflammatory response in AP mice

Male healthy C57BL/6 mice were randomly divided into two groups: AP and LysoPC + AP (Figure S9A). Compared with AP mice, LysoPC pretreatment significantly exacerbated the pancreatic injury in AP mice, leading to an increased MPO level in the pancreas (Figure S9B). Meanwhile, we found that LysoPC could elevate serum lipase and amylase activities, and intensify the inflammatory response in AP mice (Figure S9C–F). In addition, LysoPC could lead to increased gut permeability in AP mice (Figure S9G–I). LysoPC is a phospholipid by‐product of phosphatidylcholine (PC) under the catalysis of PLA2, and it has been proved to induce necrosis in rat pancreatic AR42J cells [19]. Another study demonstrated that PLA2 can exacerbate rat AP and lung injury by increasing LysoPC [20]. These findings suggest that LysoPC can aggravate the severity of AP mice.

## CONCLUSION

In conclusion, our study reveals that the HTG‐modulated gut microbiota can promote glycerophospholipid metabolism and increase LysoPC content in a TLR4‐dependent manner, thereby aggravating pancreatic injury in AP.

## METHODS

Detailed experimental materials and procedures, including sample collection and processing techniques, and statistical analysis approaches are available in the Supplementary Material.

## AUTHOR CONTRIBUTIONS

**Xiaofan Song**: Conceptualization; methodology; software; data curation; formal analysis; Writing—review and editing; Writing—original draft; funding acquisition; investigation. **Lei Qiao**: Conceptualization; methodology. **Xina Dou**: Conceptualization; methodology. **Jiajing Chang**: Writing—review and editing. **Xiaonan Zeng**: Conceptualization; project administration. **Tianjing Deng**: Investigation. **Ge Yang**: Investigation. **Peiyun Liu**: Validation. **Cheng Wang**: Conceptualization; methodology; investigation; resources. **Qinhong Xu**: Conceptualization; methodology; software; data curation. **Chunlan Xu**: Conceptualization; methodology; software; data curation; supervision; resources; project administration; formal analysis; validation; investigation; funding acquisition.

## CONFLICT OF INTEREST STATEMENT

The authors declare no conflicts of interest.

## Supporting information


**Figure S1.** HTG aggravated pancreatic injury, inflammatory response and gut barrier dysfunction in AP mice.
**Figure S2.** HTG can aggravate the damage of intestinal barrier function in AP mice.
**Figure S3.** HTG could result in gut microbiota and host metabolism dysbiosis.
**Figure S4.** Depletion of HTG‐modulated gut microbiota alleviated the pancreatic injury of AP.
**Figure S5.** Depletion of HTG‐modulated gut microbiota alleviated the damage of intestinal barrier function in HTG + AP.
**Figure S6.** HTG‐modulated gut microbiota aggravated the pancreatic injury of AP.
**Figure S7.** HTG‐modulated gut microbiota can aggravate the damage of intestinal barrier function in AP mice.
**Figure S8.** HTG‐modulated gut microbiota could regulate LysoPC content.
**Figure S9.** LysoPC aggravated pancreatic injury, inflammatory response and gut barrier dysfunction in AP mice.

**Table S1.** Mouse primer sequences for real‐time qRT‐PCR.

## Data Availability

The data that support the findings of this study are openly available in data at https://github.com/Bio-researcher/data. The raw sequence data of 16S rRNA sequencing reported in the study have been deposited in the NCBI (GSA: PRJNA1046265, https://www.ncbi.nlm.nih.gov/bioproject/PRJNA1046265/; PRJNA1046254, https://www.ncbi.nlm.nih.gov/bioproject/?term=PRJNA1046254). Partial microbiome data were analyzed on the online tool of Majorbio Cloud Platform (https://cloud.majorbio.com/page/tools/). The data used are saved in GitHub https://github.com/Bio-researcher/data. Supplementary materials (methods, figures, tables, graphical abstract, slides, videos, Chinese translated version, and update materials) may be found in the online DOI or iMeta Science http://www.imeta.science/.
