# Supplementary material for: Hypertriglyceridemia‐modulated gut microbiota promotes lysophosphatidylcholine generation to aggravate acute pancreatitis in a TLR4‐dependent manner
Source: Imeta. 2025 Feb 11;4(1):e70003. doi: 10.1002/imt2.70003 (PMC11865321; doi:10.1002/imt2.70003)
Supplement: Supplementary file 1 — Figure S1. HTG aggravated pancreatic injury, inflammatory response and gut barrier dysfunction in AP mice. Figure S2. HTG can aggravate the damage of intestinal barrier function in AP mice. Figure S3. HTG could result in gut microbiota and host metabolism dysbiosis. Figure S4. Depletion of HTG‐modulated gut microbiota alleviated the pancreatic injury of AP. Figure S5. Depletion of HTG‐modulated gut microbiota alleviated the damage of intestinal barrier function in HTG + AP. Figure S6. HTG‐modulated gut microbiota aggravated the pancreatic injury of AP. Figure S7. HTG‐modulated gut microbiota can aggravate the damage of intestinal barrier function in AP mice. Figure S8. HTG‐modulated gut microbiota could regulate LysoPC content. Figure S9. LysoPC aggravated pancreatic injury, inflammatory response and gut barrier dysfunction in AP mice. [file IMT2-4-e70003-s001.docx]

Supporting information to

Hypertriglyceridemia-modulated gut microbiota promotes lysophosphatidylcholine generation to aggravate acute pancreatitis in a TLR4-dependent manner

Running Title: HTG-modulated gut microbiota aggravates acute pancreatitis

Xiaofan Song^1^, Lei Qiao^1^, Xina Dou^1^, Jiajing Chang^1^, Xiaonan Zeng^1^, Tianjing Deng^1^, Ge Yang^1^, Peiyun Liu^1^, Cheng Wang^3^[[1]](#footnote-1)^*^ , Qinhong Xu^2^[[2]](#footnote-2)^*^, Chunlan Xu^1, 2^[[3]](#footnote-3)^*^

^1^School of Life Sciences, Northwestern Polytechnical University, Xi’an 710072, China.

^2^Department of Geriatric Surgery, the First Affiliated Hospital of Xi’an Jiaotong University, Xi’an 710061, China.

^3^Xianghu Laboratory, Hangzhou 311231, China.

*Corresponding authors: clxu@nwpu.edu.cn (Chunlan Xu); xuqinhong8410@163.com (Qinhong Xu); wangcheng@xhlab.ac.cn (Cheng Wang)

**MATERIALS AND METHODS**

**AP Mouse Models with or without HTG**

Male healthy C57BL/6 wild-type mice (4 weeks old) and *Tlr4*^-/-^ mice (male, 4 weeks old) were purchased from Beijing Huafukang Biological Technology Co., LTD (Beijing, China) and housed under standard conditions (temperature: 25 ± 2 ℃; relative humidity, 50 ± 5 %; and a 12/12 h light/dark cycle) for 1 week acclimatization before formal experiment were performed. Mice had ad libitum access to drinking water and standard diet throughout the experimental period. All animal studies were approved by the Ethics Committee of Northwestern Polytechnical University, China (No. 20220721). At the end of the experiment, mice were sacrificed after a 12 h fast, and eyeball blood, pancreatic tissue and cecal contents were rapidly collected in a sterile environment. Samples were packed and stored at −80°C until analysis. The mice model of hypertriglyceridemic acute pancreatitis (HTG + AP) was induced by a combination of caerulein (TAIJIA Biotech Co., Ltd, Hangzhou, China) and Poloxamer-407 (Pluronic F-127, Sigma-Aldrich, Saint-Louis, Missouri, USA).

**Germ-free Mouse Models and Fecal Microbiota Transplantation**

20 4-week-old male C57BL/6 mice were randomly divided into 2 groups: antibiotic-treated HTG + AP (Abx-HTG + AP) mice and HTG + AP mice, with 10 mice in each group. The Abx-HTG + AP mice were administered broad-spectrum antibiotics (50 μg/mL clindamycin, 50 μg/mL metronidazole, 50 μg/mL penicillin and 50 μg/mL neomycin) in drinking water for 2 weeks. The HTG + AP mice were given normal water for 2 weeks, after which HTG + AP models were constructed as described above. Germ-free mice were kept drinking broad-spectrum antibiotics during model construction; In addition, in the fecal microbiota transplantation experiment (FMT), 20 4-week-old male C57BL/6 mice were randomly divided into 2 groups: mice received Control fecal microbiota (FMT-Control + AP) and mice received HTG fecal microbiota (FMT-HTG + AP), with 10 mice in each group. 150mg of the fecal samples from HTG and Control mice were collected and placed in 1mL of normal saline, respectively. The collected samples were shaken evenly and centrifuged at 2500 rpm for 5min at 4 °C for a total of two times. After that, the supernatant was centrifuged at 8000 rpm for 5min at 4 °C, the pellet was resuspended in 1mL of phosphate buffer solution (PBS), and 50% sterile glycerol was added to the final concentration of 20%, and then stored at −80 °C. 200 uL fecal microbiota was gavaged in each germ-free recipient for 8 weeks. After this, the AP mouse model was established as described above.

**LysoPC treatment of mice**

To investigate the role of lysophosphatidylcholine (LysoPC) in the pathogenesis of AP, male healthy C57BL/6 mice (4 weeks old) were randomly divided into two groups (AP group and LysoPC + AP group). Before the AP model was established, LysoPC + AP mice were treated with LysoPC (40 mM, dissolved in saline) by gavage for 4 weeks, and AP mice were treated with the same amount of saline for 4 weeks. And then AP models were established as described above. After 12 h, the mice were sacrificed, and serum and tissue samples were collected for subsequent experiments.

**Isolation and treatment of pancreatic acini**

After fasting for 12 h, male healthy C57BL/6 mice were sacrificed, the pancreas was removed, and pancreatic acinar cells were isolated by collagenase digestion. Afterwards, the isolated cells were resuspended in DEME/F12 (HAM) 1:1 medium supplemented with 10% FBS and 1% antibiotics mixture (100 U/mL penicillin and 100 μg/mL streptomycin). During the experiment, the cells were divided into Control group, lipopolysaccharides (LPS) group and toll-like receptor 4 (TLR4) inhibitor (TAK-242) + LPS group. For TAK-242 + LPS group, the pancreatic acinar cells were pretreated with TAK-242 at a final concentration of 100 nM for 4 h. The cells of Control group and LPS group were treated with the same volume of PBS. For LPS and TAK-242 + LPS groups, cells were exposed to 2 μg/mL LPS for 24 h. After the above treatments, the cell supernatant and protein homogenate were collected for subsequent experiments.

**Biochemical detection**

Serum was obtained by centrifuging whole blood samples. The pancreas and hepatic were harvested for preparation of homogenate. The levels of tumour necrosis factor-alpha (TNF-α) and interleukin-1 beta (IL-1β) were detected by ELISA kits (Jianglaibio, Shanghai, China) according to the manufacturer’s instruction. The activities of amylase and lipase in serum were measured by the corresponding kits (Solarbio, Beijing, China) according to the manufacturer's instructions. The levels of total cholesterol (TC), triglycerides (TG) and free fatty acids (FFAs) were detected by the corresponding kits (Solarbio, Beijing, China). The endotoxin, phospholipase A2 (PLA2) and LysoPC were detected by the corresponding ELISA kits (Hengyuan biological, Shanghai, China).

**Identification of bacterial translocation**

Whole blood and pancreas of mice were collected by aseptic operation. MacConkey media (Haibo, Qingdao, China) were used for gram-negative and colistin/nalidixic acid (CNA) 5% sheep blood agar (Haibo, Qingdao, China) for gram-positive bacteria. The plates were incubated at 37 ℃ for 24 h, and the number of monoclonals in the plates was counted. Colony forming units (CFU) in blood and pancreas was detected by viable counting method.

**Immunohistochemistry and Histological analysis**

Pancreas histology was assessed by H&E staining of paraffin-embedded sections. The expression levels of MPO in the pancreas was assessed by immunohistochemistry.

**Western Blot analysis**

Total protein was isolated from pancreas and gut by the RIPA buffer containing protease inhibitor cocktail. The protein concentration was determined by the BCA Protein Assay kit (AccuRef Scientific, Xi’an, China). Individual sample protein concentrations were adjusted to homogenization and boiled in loading buffer for 15 min. After that, equal volumes and equal amounts of samples were loaded into SDS-PAGE gel, and then transferred to the PVDF membrane. The membranes were incubated with zona occludens 1 (ZO-1) (ABclonal, Wuhan, China), occludin (OCLD) (ABclonal, Wuhan, China), claudin-1 (CLDN1) (ABclonal, Wuhan, China), toll-like receptor 4 (TLR4) (ABclonal, Wuhan, China), myeloid differentiation primary response protein 88 (MyD88) (ABclonal, Wuhan, China), nuclear factor kappa-B (NF-κB) (Proteintech, Wuhan, China), phospho-nuclear factor kappa-B (p-NF-κB) (Proteintech, Wuhan, China), inhibitor of nuclear factor kappa-B (IκB) (Proteintech, Wuhan, China), phospho-inhibitor of nuclear factor kappa-B (p-IκB) (Proteintech, Wuhan, China) and β-actin (Proteintech, Wuhan, China) primary antibodies overnight at 4 ℃. Then the membranes were incubated with secondary antibodies (Servicebio, Wuhan, China) for 1 h at room temperature. Immunoreactive protein bands were visualized by the clarity Western ECL substrate kit and detected by Tanon 5200 Multi (Shanghai, China). Finally, Image-J analysis software (National Institute of Health, Bethesda, MD, USA) was applied to quantitatively analyze each protein band.

**Quantification RT-PCR**

Total RNA was extracted using the TRIzol Reagent (Servicebio, Wuhan, China). RNA concentration was determined by a nanophotometer (Stuttgart, Germany) and was reverse transcribed by a complementary DNA conversion kit (Genesand, Beijing, China). qRT-PCR was performed using SYBR Green Master Mix (Genesand, Beijing, China) in the Line-Gene 9600 Plus Real-Time PCR System (Shanghai, China). The specific primers of tested genes were listed in the Table S1.

**Bioinformatics analysis of gut microbiota**

The microbial DNA was extracted from cecal contents samples of C57BL/6 mice using the Fast DNA Spin Kit (MP Biomedicals, US). Total DNA mass was determined using a NanoDrop 2000 spectrophotometer (Thermoelectric Science, Massachusetts, USA) and 1% agarose gel electrophoresis (AGE). The forward primer 338F (5’-ACTCCTACGGGAGGCAGCAGCAG-3’) and reverse primer 806R (5’ -GGACTACHVGGGTWTCTAAT-3’) were used to amplify the V3-V4 variable region of 16S rRNA gene. Purified amplicons were paired sequenced on an Illumina MiSeq PE300 platform (Illumina, San Diego, USA). Then, the data were optimized by noise reduction to obtain the Amplicon Sequence Variants (ASVs) representing the sequence and abundance information. Based on silva138/16s_bacteria species taxonomic analysis of ASVs using the Naive bayes classifier in QIIME2.

**Bioinformatics analysis of liquid chromatography-mass spectrometry (LC-MS) Untargeted Metabolomics**

100 µL serum was mixed thoroughly with 400 µL methanol: water (4:1, v/v) solution. The mixture was allowed to settle at -20 ℃ and treated with high throughput tissue crusher Wonbio-96c (Shanghai wanbo biotechnology co., LTD) at 50 Hz for 6 min. The samples were allowed to stand at -20 ℃ for 30 min and centrifuged at 13,000 g, 4 ℃ for 15 min. After that, the supernatant was aspirated into the sample vial for LC-MS analysis. Equal volumes were taken from each prepared sample as quality control samples, and a quality control sample was inserted into every 4 samples. The instrument platform for this LC-MS analysis was UPLCTripleTOF system of AB SCIEX ultra performance liquid chromatography tandem time of flight mass spectrometry.

**Statistical analyses**

GraphPad Prism V.8 (Graphpad software, San Diego, California, USA) or SPSS 26.0 was used to analyze the data. Unless otherwise stated, results were expressed as mean ± standard deviation (SD). For comparisons between two groups, non-parametric Mann-Whitney U test or Student’s *t*-test was used to test statistical significance, depending on the distribution of normality. Analysis of variance (ANOVA) and Tukey's test were used to test for differences between multiple groups. Fisher exact test was used to analyze the difference between the positive results of bacterial culture of whole blood and pancreatic tissue. Permutation multivariate ANOVA (PERMANOVA) was used for group-wise comparisons of β-diversity.

**Figure S1** **HTG aggravated pancreatic injury, inflammatory response and gut barrier dysfunction in AP mice.** (A) Representative gross morphology and microscopic features in pancreas of mice (*n* = 3, Scale bars: 100 µm). (B) Schematic illustration of the treatment of C57BL/6 mice (*n* = 10). (C-E) Serum TG (C), TC (D) and FFAs (E) contents in mice (*n* = 6). (F) FFAs content in mice pancreas (*n* = 6). (G) Survival rate of mice (*n* = 10). (H) Serum amylase activity (*n* = 6). (I) Serum lipase activity (*n* = 6). (J) Representative microscopic features and immunohistochemical staining of MPO in the pancreas of mice (*n* = 3, Scale bars: 100 µm). (K) Serum IL-1β level (*n* = 6). (L) Serum TNF-α level (*n* = 6). ^*^*p* < 0.05, ^**^*p* < 0.01, ^***^*p* < 0.001. HTG, hypertriglyceridemia; AP, acute pancreatitis; HTG + AP, hypertriglyceridemic acute pancreatitis; TG, triglyceride; TC, total cholesterol; FFAs, free fatty acids; MPO, myeloperoxidase; IL-1β, interleukin-1 beta; TNF-α, tumour necrosis factor-alpha.

**Figure S2** **HTG can aggravate the damage of gut barrier function in AP mice.** (A) Tight junction proteins expression levels in mice cecum (*n* = 3). (B-D) Serum DAO (B), D-LA (C) and endotoxin (D) in mice (*n* = 6). (E and F) Bacterial density in the pancreas (E) or blood (F) of mice (*n* = 10). (G) Frequency of culture positivity in blood and pancreas (*n* = 10). ^*^*p* < 0.05, ^**^*p* < 0.01, ^***^*p* < 0.001. HTG, hypertriglyceridemia; AP, acute pancreatitis; HTG + AP, hypertriglyceridemic acute pancreatitis; DAO, diamine oxidase; D-LA, D-lactic acid; ZO-1, zona occludens 1; OCLD, occludin; CLDN1, claudin-1.

**Figure S3** **HTG could result in gut microbiota dysbiosis.** (A) The ACE index of the gut microbiota (*n* = 6). (B) The Shannon index of the gut microbiota (*n* = 6). (C) The Simpson index of the gut microbiota (*n* = 6). (D) The observed_species of the gut microbiota (*n* = 6). ^*^*p* < 0.05. HTG + AP, hypertriglyceridemic acute pancreatitis; Abx, antibiotic treatment; ACE, abundance-based coverage estimator.

**Figure S4** **Depletion of HTG-modulated gut microbiota alleviated the pancreatic injury of AP.** (A) Schematic illustration of the treatment of C57BL/6 mice (*n* = 10). (B) The Ace index of the gut microbiota (*n* = 6). (C) The Chao index of the gut microbiota (*n* = 6). (D) The Shannon index of the gut microbiota (*n* = 6). (E) Representative microscopic features and immunohistochemical staining of MPO in the pancreas of mice (*n* = 3, Scale bars: 100 µm). (F and G) Serum TNF-α (F) and IL-1β (G) levels (*n* = 6). (H) Serum amylase activity (*n* = 6). (I) Serum lipase activity (*n* = 6). (J-L) Serum TG (J), TC (K) and FFAs (L) contents in mice (*n* = 6). (M) FFAs content of mice pancreas (*n* = 6). ^*^*p* < 0.05, ^**^*p* < 0.01, ^***^*p* < 0.001. HTG + AP, hypertriglyceridemic acute pancreatitis; Abx, antibiotic treatment; TG, triglyceride; TC, total cholesterol; FFAs, free fatty acids; MPO, myeloperoxidase; ACE, abundance-based coverage estimator; IL-1β, interleukin-1 beta; TNF-α, tumour necrosis factor-alpha.

**Figure S5 Depletion of HTG-modulated gut microbiota alleviated the damage of intestinal barrier function in HTG+AP.** (A) Tight junction proteins expression levels in the mice cecum (*n* = 3). (B-D) Serum DAO (B), D-LA (C) and endotoxin contents (D) in mice (*n* = 6). (E and F) Bacterial density in the blood (E) or pancreas (F) of mice (*n* = 10). (G) Frequency of culture positivity in blood and pancreas (*n* = 10). ^*^*p* < 0.05, ^***^*p* < 0.001. HTG + AP, hypertriglyceridemic acute pancreatitis; Abx, antibiotic treatment; DAO, diamine oxidase; D-LA, D-lactic acid; CFU, colony forming units; ZO-1, zona occludens 1; OCLD, occludin; CLDN1, claudin-1.

**Figure S6** **HTG-modulated gut microbiota aggravated the pancreatic injury of AP.** (A) Schematic illustration of the treatment of C57BL/6 mice (*n* = 10). (B) Representative microscopic features and immunohistochemical staining of MPO in the pancreas of mice (*n* = 3, Scale bars: 100 µm). (C) Serum amylase activity (*n* = 6). (D) Serum lipase activity (*n* = 6). (E and F) Serum IL-1β (E) and TNF-α (F) levels (*n* = 6). (G-I) Serum TG (G), TC (H) and FFAs (I) contents in mice (*n* = 6). (J) FFAs content of mice pancreas (*n* = 6). ^*^*p* < 0.05, ^**^*p* < 0.01, ^***^*p* < 0.001. HTG + AP, hypertriglyceridemic acute pancreatitis; FMT, fecal microbiota transplantation; TG, triglyceride; TC, total cholesterol; FFAs, free fatty acids; MPO, myeloperoxidase; IL-1β, interleukin-1 beta; TNF-α, tumour necrosis factor-alpha.

**Figure S7** **HTG-modulated gut microbiota can aggravate the damage of gut barrier function in AP mice.** (A) Tight junction proteins expression levels in the mice cecum (*n* = 3). (B-D) Serum DAO (B), D-LA (C) and endotoxin contents (D) in mice (*n* = 6). (E and F) Bacterial density in the blood (E) or pancreas (F) of mice (*n* = 10). (G) Frequency of culture positivity in blood and pancreas (*n* = 10). ^*^*p* < 0.05, ^**^*p* < 0.01, ^***^*p* < 0.001. HTG + AP, hypertriglyceridemic acute pancreatitis; FMT, fecal microbiota transplantation; DAO, diamine oxidase; D-LA, D-lactic acid; CFU, colony forming units; ZO-1, zona occludens 1; OCLD, occludin; CLDN1, claudin-1.

**Figure S8 HTG-modulated gut microbiota could regulate LysoPC content.** (A) The content of PLA2 in serum (*n* = 6). (B) Lipid metabolism-related genes mRNA levels in pancreas (*n* = 6). (C and D) The LysoPC content in serum (C) and pancreas (D) (*n* = 6). (E) The content of PLA2 in serum (*n* = 6). (F) Lipid metabolism-related genes mRNA levels in pancreas (*n* = 6). (G and H) The contents of endotoxin in cecal contents (G) and pancreas (H) (*n* = 6). (I) The effect of HTG-modulated gut microbiota on TLR4 (*n* = 3). (J and K) The contents of endotoxin in cecal contents (J) and pancreas (K) (*n* = 6). (L) The effect of HTG-modulated gut microbiota on TLR4 signaling pathway (*n* = 3). ^*^*p* < 0.05, ^**^*p* < 0.01. HTG + AP, hypertriglyceridemic acute pancreatitis; Abx, antibiotic treatment; LysoPC, lysophosphatidylcholine; PLA2, phospholipase A2; *Pla2g2a*, phospholipase A2 group IIA; *Pla2g4a*, phospholipase A2 group IVA; *Lcat,* lecithin cholesterol acyltransferase; FMT, fecal microbiota transplantation; TLR4, toll-like receptor 4; MyD88, myeloid differentiation primary response protein 88; NF-κB, nuclear factor kappa-B; p-NF-κB, phospho-nuclear factor kappa-B; IκB, inhibitor of nuclear factor kappa-B; p-IκB, phospho-inhibitor of nuclear factor kappa-B.

**Figure S9** **LysoPC aggravated pancreatic injury, inflammatory response and gut barrier dysfunction in AP mice.** (A) Schematic illustration of the treatment of C57BL/6 mice (*n* = 10). (B) Representative microscopic features and immunohistochemical staining of MPO in pancreas of mice (*n* = 3, Scale bars: 100 µm). (C) Serum lipase activity (*n* = 6). (D) Serum amylase activity (*n* = 6). (E and F) Serum IL-1β (E) and TNF-α (F) levels (*n* = 6). (G-I) Serum DAO (G), D-LA (H) and endotoxin contents (I) in mice (*n* = 6). LysoPC, lysophosphatidylcholine; DAO, diamine oxidase; D-LA, D-lactic acid; MPO, myeloperoxidase; AP, acute pancreatitis; IL-1β, interleukin-1 beta; TNF-α, tumour necrosis factor-alpha.

1. [↑](#footnote-ref-1)
2. [↑](#footnote-ref-2)
3. [↑](#footnote-ref-3)
